# Time to recovery from severe community-acquired pneumonia and its predictors among 6 to 59 months of age children admitted to South Wollo zone public hospitals, North East Ethiopia: a prospective follow-up study

**DOI:** 10.1186/s41479-024-00135-x

**Published:** 2024-08-05

**Authors:** Mekonnen Teferi, Elsabeth Addisu, Shambel Wodajo, Amare Muche, Abel Endawekie, Bezawit Adane, Tilahun Dessie, Natnael Kebede

**Affiliations:** 1 Department of Biostatistics and Epidemiology, Kemisse Health Sciences College, Kemisie, Ethiopia; 2 Department of reproductive and family health, School of Public Health, College of Medicine and Health Sciences, Wollo University, Dessie, Ethiopia; 3 Department of Biostatistics and Epidemiology, School of public health, College of Medicine and Health Sciences, Wollo University, Dessie, Ethiopia; 4 Department of Pediatrics and child health, School of Medicine, College of Medicine and Health Sciences, Wollo University, Dessie, Ethiopia; 5 Department of Health Promotion, School of public health, College of Medicine and Health Sciences, Wollo University, Dessie, Ethiopia

**Keywords:** Time to recovery, Severe community-acquired pneumonia, South Wollo

## Abstract

**Introduction:**

Ethiopia is one of those countries with higher burden of community acquired pneumonia among its people, under five children are the members of society that are highly affected by pneumonia particularly Severe Community Acquired Pneumonia. However, there are limited studies on time to recovery and its predictors in under-five children and most of them are retrospective which fails to address important variables that affect the time to recovery. Therefore, the aim of this study was to estimate the median time to recovery and its predictors among under five children admitted to South Wollo zone public hospitals, North East Ethiopia.

**Methods:**

An institution-based prospective cohort study was conducted from March 10 to May 10, 2021, with 270 study subjects. A systematic random sampling technique was used. Data was collected by interview and chart review. The data were entered and analyzed using Epi Data version 3.1 and STATA version 14.0, respectively. Kaplan-Meier and Cox regression models were used to test the time and predictors of recovery from severe community-acquired pneumonia.

**Results:**

The overall incidence of recovery rate (95% confidence interval) from Severe Community-Acquired Pneumonia was 20.45(17.84–23.46) per 100 person days observation with median (IQR) time to recovery of [3, 5] days. The predictors of time to recovery from Severe Community-Acquired Pneumonia were having comorbidities on admission [AHR = 0.49 (95%CI: 0.32,0.75)], reaching hospitals after 5 days of onset of symptoms [AHR = 0.35 (95%CI: 0.20,0.60)], having Middle Upper Arm Circumference < = 12.5 cm [AHR = 0.21 (95%CI: 0.12,0.37)], the presence of smoker in the house [AHR = 0.21 (95%CI: 0.10,0.42)] and being not fully immunized for age [AHR = 0.35 (95%CI: 0.24,0.53)].

**Conclusion and recommendations:**

Generally the recovery time of children with Severe Community Acquired Pneumonia in the study area was within the recommended national standards. Due attention should be given to children with the identified predictors while treating them.

## Introduction

Pneumonia is an acute respiratory tract infection (ARTI) that affects the parenchymal tissues of the lungs [1]. During normal breathing, small sacs in the lungs called alveoli fill with air. When children contract pneumonia the alveoli fill with pus and fluid, restricting breathing and making it painful [2].

Globally, pneumonia is a major cause of morbidity and mortality among children which leads to over 100 million episodes and 9 million hospitalizations each year and it is a substantial cause of childhood morbidity and mortality in developing countries [3]. About 20% of all deaths in children under five years of age have been reported to be happened due to acute lower respiratory infection (ALRI) which includes: pneumonia, bronchiolitis, and bronchitis. About 90% of ALRI-associated deaths take place due to severe pneumonia [4].

Pneumonia can be classified as community-acquired pneumonia and hospital-acquired pneumonia. Community-acquired pneumonia (CAP) is an infection that begins outside the hospital or is diagnosed within 48 h after admission to the hospital in a person who has not resided in a long-term care facility for 14 days or more before admission [5]. Hospital-acquired pneumonia is pneumonia that occurs more than 48 h after admission and without any antecedent signs of infection at the time of hospital admission [6]. CAP is a leading infectious disease requiring hospital admission and constitutes a major burden on health care resources [7] .

Pneumonia can also be classified into three phases based on the severity of its clinical presentation which are distinguishable by the use of physical examination findings. These are no pneumonia (cough or cold), pneumonia, and severe pneumonia [8].

In developing countries, respiratory tract infections are not only more prevalent but severe also, approximately 13% of pneumonia cases are severe enough to require hospitalization [9]. Of all the pneumonia cases occurring in countries with high incidence, 8.7% are severe enough to be life-threatening [10].

Severe pneumonia in childhood is associated with increased long-term respiratory morbidity and disease burden and is more fatal than non-severe disease [11]. Childhood pneumonia remains a leading killer of children globally, where it accounts for up to 15% of deaths in children under the age of five years [12].

A report from the US Centers for Diseases Control and Prevention estimated that the average length of hospital stay for treatment of pneumonia in children aged < 15 years (excluding neonates) is 5 days. Any hospital stay exceeding 5 days is considered to be prolonged [13]. Those factors which are associated with prolonged hospital stay include increased age of the child [14], having a smoker in the house [15, 16], late presentation to seek care [14], presence of comorbidities at admission such as head nodding, the presence of oedematous Protein-energy malnutrition, severe wasting, and hypoxemia at presentation [16], mothers education less than Secondary school graduation and lack of exclusive breast feeding [17].

Pneumonia has been reduced significantly after the introduction of the pneumococcal conjugate vaccine (PCV) and it can also be easily treated with low-cost antibiotics if properly diagnosed but tens of millions of children are still going unvaccinated and one in three with symptoms will not receive essential medical care [9]., pneumonia is still a major public health problem for children, especially in developing countries [4]. Studies were conducted on the prevalence, associated factors, and determinants of pneumonia among under-five children. However, those studies did not determine the predictors of recovery time. Recovery time and its predictors of children’s hospitalization related to SCAP are not well known. Additionally, since most of the researches were conducted retrospectively from chart review, these studies failed to include important variables like socio demographic and economic status of the care givers which could affect the recovery time of children with SCAP. Therefore the aim of this study is to determine time to recovery from SCAP and its predictors among 6 to 59 months of age children admitted to South Wollo zone public hospitals North East Ethiopia.

## Materials and methods

### Study setting, study design, and population

This study was conducted from March 10 to May 10, 2021 at public hospitals of South Wollo zone, which is one of the 14 zones of Amhara region. The capital of the South Wollo zone is Dessie city which is located 401 km from Addis Ababa in the north east of Ethiopia. There are a total of 13 public hospitals at South Wollo zone serving about 3 to 4 million People of which 10 of them are primary hospitals, 2 general hospitals and 1 specialized hospital. An institution-based prospective follow-up study was conducted. All children from 6 to 59 month of age who have caregiver and admitted to South wollo zone public hospitals with severe CAP during the study period were included in this study.

### Sample size determination and procedure

The sample size was calculated for the Cox model by considering the probability of time to recovery 0.89, probability of withdrawal 0.051, 95%CI, power 80% and Adjusted Hazard Ratio (AHR) of 0.69 for a child admitted with danger sign predictors which have a significant association with time to recovery [14]. It was calculated using STATA software and the final sample size for this study was 270.

From the total of 13 hospitals in the South Wollo zone, study subjects were proportionally allocated and selected using a systematic random sampling method. The total number of monthly under-5 admissions in those hospitals is estimated to be 720 and the total number of monthly under-5 admission due to SCAP is estimated to be 264 based on the last three months’ admission profile of each hospital. We have implemented a systematic sampling method for this study and calculated the value of K, which represents the interval between selected participants, and determined it to be two. The random start was selected randomly. The study participants were selected for two individuals based on their order of admission taken from the ward admission record.

### Variables measurement

#### Dependent variable

Time to recovery from severe community-acquired pneumonia.

#### Independent variables

- Age
- EBF
- Immunization status
- Parents’ educational status and occupation
- Smoker in the house
- Duration to seek care
- Family size
- Total number of under-five children
- Presence of concomitant disease (co-morbidities) and complications on admission (Malnutrition and danger signs)
- Level of health facility
- Number of trained staff in the Pneumonia treatment center
- Total number of children admitted with SCAP

### Operational definitions

#### Event

recovery from severe community-acquired pneumonia during the study period [14].

#### Recovery

children improved from SCAP as declared by the clinician/physician [14].

#### Death

a patient who died while he/she was being treated in the program in a facility [14].

#### Defaulter

is a SCAP patient that was absent from the hospital for two consecutive days [14].

#### Non- respondent

A patient who could not meet the discharge criteria after five days of inpatient management [14].

#### Censored

children referred to facilities located outside the study area, died, defaulted, or respondent [14].

#### Duration to seek care

duration in days from onset of symptoms to health facility visit. Those who presented for treatment within 5 days of the onset of symptoms were classified as **early presenters**, while those who presented after 5 days were **late presenters** [18].

#### Co-morbidity

any disease condition (acute or chronic) present at admission in addition to SCAP [14].

#### Danger signs

loss of consciousness, abnormal body movement, vomiting of everything, convulsions, and inability to feed in addition to SCAP [14].

#### Immunization status

**fully immunized** was defined as children who had completed all forms of vaccinations expected for his/her age; **not fully immunized** was defined as children who missed at least 1 of the immunizations expected for his/her age which is confirmed by checking their Immunization card or by asking the care giver the number and timing the child takes vaccines [14].

#### Smoker in the house

having a smoker in the house was defined as having a family member who smoke cigarette inside the house where children can live [15].

### Data collection procedure and quality control

The data were collected using a pretested structured Amharic version questionnaire adapted from previous studies. A structured data extraction check list was also prepared from standard treatment protocols for chart review. Data was collected by interviewing the care givers of the child and by reviewing daily patient records by one trained health professional (Clinical Nurse), and one BSc Nurse assigned as a supervisor for each hospital. The questionnaire was developed in English, then translated into Amharic language, and again translated back to English to ensure consistency. Data collectors and supervisors were trained for one day on the objective of the study, the content of the questionnaire, and the data collection procedure. Data were pretested on 5% of the total sample size at Woldia hospital and based on feedback obtained from the pretest, the necessary modification was performed. During the study period, the collected data were checked continuously on a daily basis for completeness.

### Data processing and analysis

The data were entered into Epi Data version 4.6 and exported to Stata/SE version 14.0 for analysis. Tables, graphs, charts, and texts were used to present descriptive data. The patient follow-up characteristics were described in terms of mean (standard deviation) and median (interquartile range) for continuous data and frequency distribution for categorical data.

To compare survival curves or to estimate time to recovery from SCAP Kaplan-Meier survival estimate was used. Both Bivariable and Multi variable Cox regression models were executed to identify predictors of recovery and those variables having P value ≤ 0.2 during Bivariable analysis were entered into the Multivariable analysis. The Cox proportional hazard assumption was checked by using the Schoenfeld residuals test and satisfied for all predictors. Adjusted Hazard Ratio with 95% CI was used to test the strength of association at a p-value of 0.05.

## Results

### Socio-demographic characteristics of children and caregivers

Of the total 270 study subjects admitted to the hospitals, 135 (50%) of children were males while 200 (74%) of children were in the age group of 6–23 months with a mean age of 17.4 months (SD ± 9.12 months). And from the parents, most of them 167 (61.85%) were at the age of 30 and below, and 214 (79.26%) of them were married (Table 1).

**Table 1:** Socio-demographic characteristics of children and caregivers, South Wollo zone, North East Ethiopia, 2021. (n = 270)

Variables	Categories	Frequency	Percent
Age of child (months)	6–11	93	34.44
	12–23	107	39.63
	24–35	47	17.41
	36–48	23	8.52
Sex of child	Male	135	50
	Female	135	50
Residence	Urban	144	53.33
	Rural	126	46.67
Age of caregivers	<=30	167	61.85
	> 30	103	38.15
Sex of care givers	Male	41	15.19
	Female	229	84.81
	Cannot read and write	97	35.93
Educational status	able to read and write	38	14.07
	educated (formal education)	98	36.30
	degree and above	37	13.70
Marital status	Married	214	79.26
	Divorced	43	15.93
	Widowed	9	3.33
	Separated	4	1.48
Occupation of caregivers	House wife	136	50.37
	Farmer	50	18.52
	Government employee	66	24.44
	Other	18	6.67

### Clinical and admission characteristics and treatment given

From a total of 270 children admitted with SCAP, 236 (87.41%) visited the hospitals within 5 days of onset of symptoms and 89 (32.96%) had previous history of ALRTI. During admission, 56 (20.74%) of children have vomiting and 163 (60.37%) of children were currently breastfeeding. Malnutrition and diarrhea followed by anemia were the common comorbidities with a prevalence of 20%, 14.81%, and 10.37% respectively (Table 2).

**Table 2:** clinical and admission characteristics of 6 to 59 months of age children with SCAP, admitted to South Wollo zone public hospitals, North East Ethiopia, 2021. (n = 270)

Characteristics	Category	Frequency	Percent
Duration to seek care	<= 5 days of symptom onset	236	87.41
	After 5 days of symptom onset	34	12.59
Comorbidities	Present	108	40
	Absent	162	60
Previous history of ALRTI	Yes	89	32.96
	No	181	67.04
Malnutrition	No malnutrition	216	80.00
	MAM	39	14.44
	SAM	15	5.56
Vomiting	Present	56	20.74
	Absent	214	79.26
Diarrhea	Present	40	14.81
	Absent	230	85.19
HIV status of the child	Negative	48	17.78
	Unknown	222	82.22
Does the child have TB	No	124	45.93
	Unknown	146	54.07
Does the child have Malaria	No	104	38.52
	Unknown	166	61.48
Anemia	Present	28	10.37
	Absent	242	89.63
Convulsion	Present	16	5.93
	Absent	254	94.07
Unconsciousness	Present	6	2.22
	Absent	264	97.78
IV fluid given	Yes	82	30.37
	No	188	69.63
IV antibiotics given	Yes	263	97.41
	No	7	2.59
Fully immunized for his/her age	Yes	188	69.63
	No	82	30.37
currently breastfeeding	Yes	163	60.37
	No	107	39.63

### Treatment outcomes of children admitted with SCAP

**Fig. 1:**
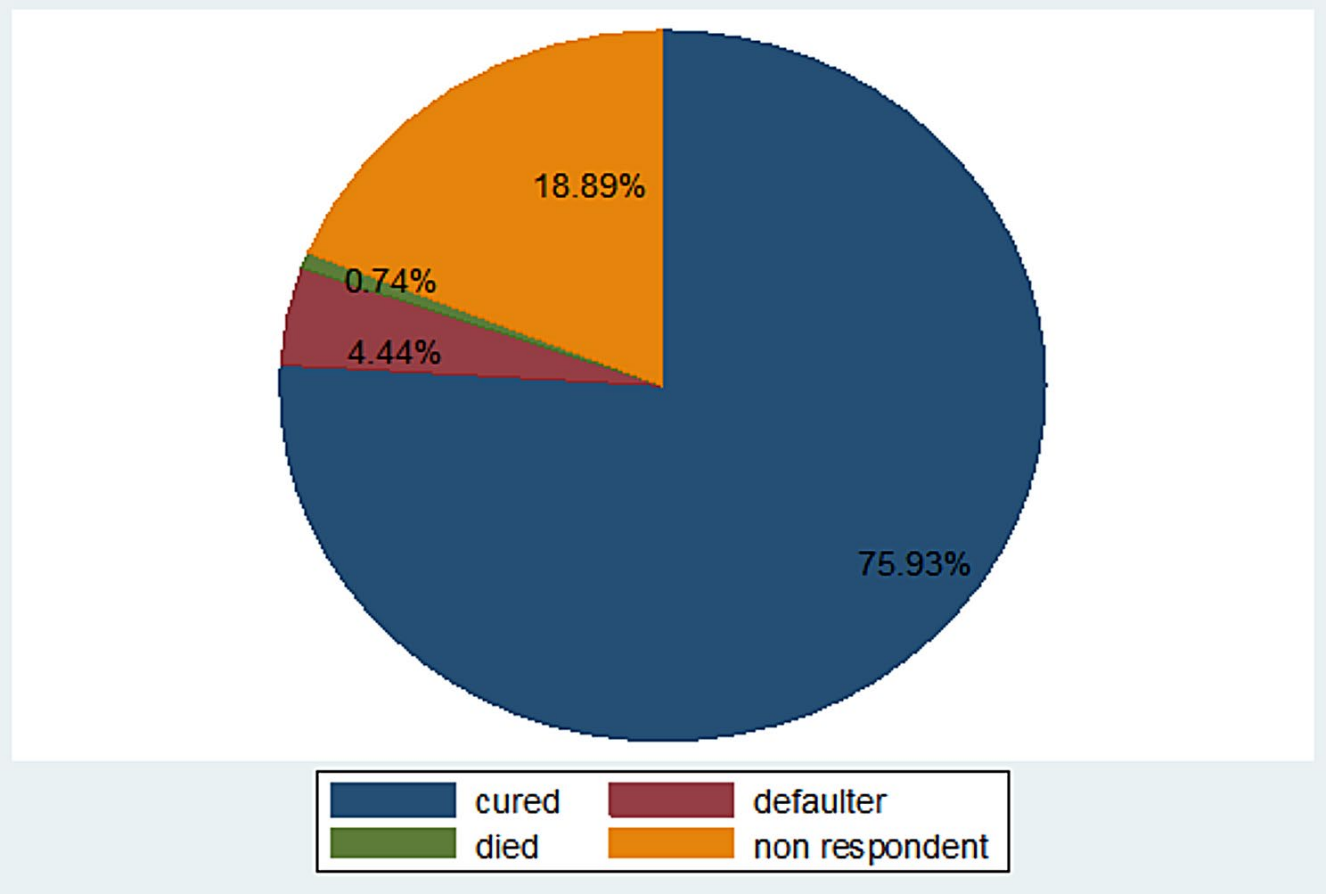
Treatment outcomes of 6 to 59 months of age children admitted with severe community acquired pneumonia in South Wollo zone public Hospitals, North East Ethiopia, 2021. (n = 270)

From the total study participants, 12 (4.44%) children were defaulted, 51 (18.89%) were not responding for treatment, 2 (0.74%) of them died and 205 (75.93%) of them were recovered from their illness. The cumulative Proportion (95% CI) of recovery during follow-up period was 0.76 (0.70, 0.81) (Fig. 1).

### Incidence and median time to recovery

The patients were followed for a minimum of 1 and a maximum of 5 days with 4 (3, 5) days median (IQR) follow-up time. The median recovery time of children from SCAP varied among various categories of socio-demographic, nutritional and clinical characteristics.

The total person-time risk was 1002. And the overall incidence of recovery rate (95%CI) from SCAP was 20.45 (95% CI: 17.84–23.46) per 100 person days observation.

### Survival estimates for time to recovery

**Fig. 2:**
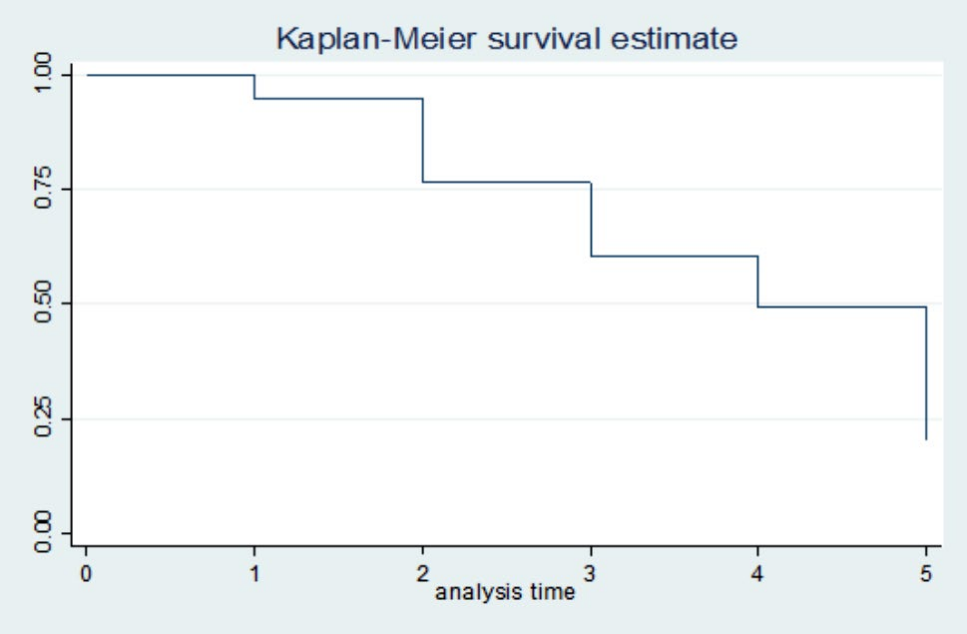
Kaplan-Meier survival estimate of recovery time among 6 to 59 months of age children with SCAP admitted to South Wollo zone public hospitals, North East Ethiopia, 2021. (n = 270)

**Fig. 3:**
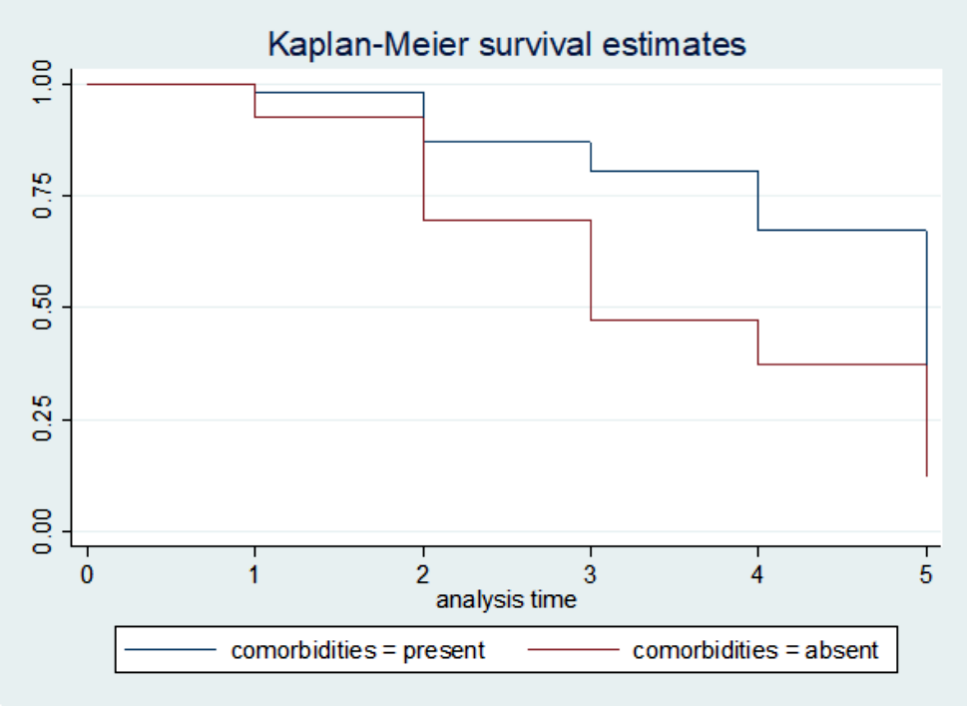
Kaplan-Meier survival estimate for time to recovery from SCAP among 6 to 59 months of age children with and without comorbidity, North East Ethiopia, 2021. (n = 270)

**Fig. 4:**
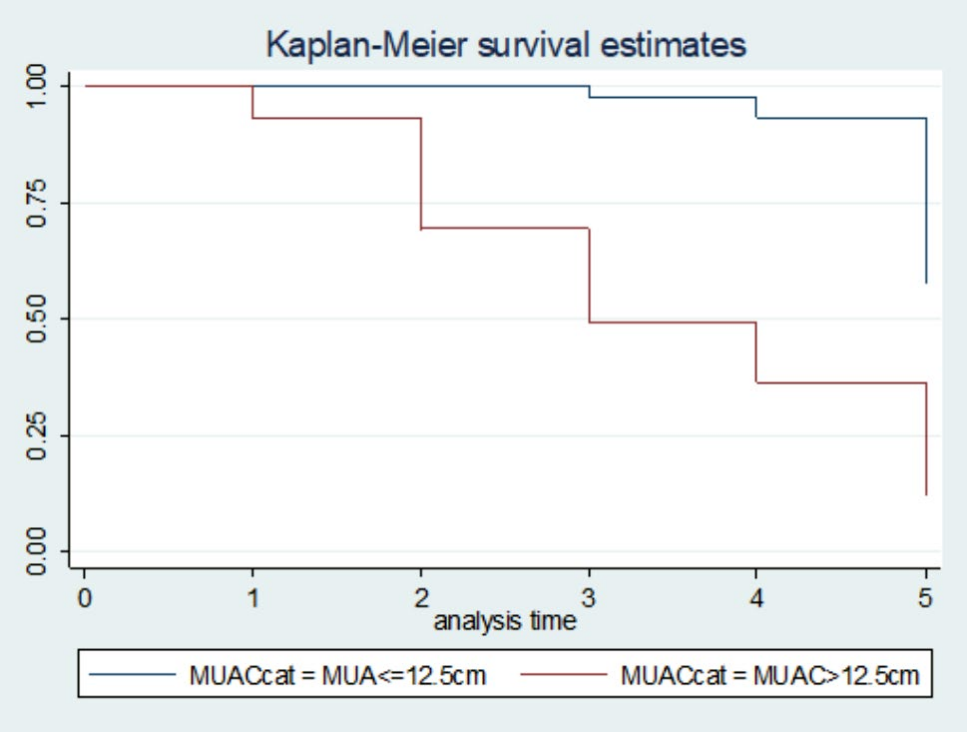
Kaplan-Meier survival estimate for time to recovery from SCAP among 6 to 59 months of age children with MUAC above and below 12.5cm, North East Ethiopia, 2021. (n = 270)

**Fig. 5:**
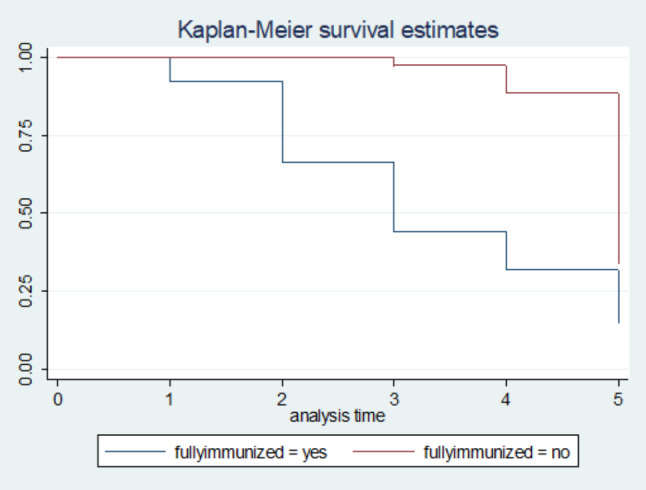
Kaplan-Meier survival estimate for time to recovery from SCAP among 6 to 59 months of age children with respect to their immunization status, North East Ethiopia, 2021. (n = 270)

The survival status of children with SCAP was estimated by the Kaplan-Meier survival curve. The curve tends to decrease rapidly at the fifth day of observation indicating that most children recovered from the disease on the fifth day (Fig. 5). The survival estimates of SCAP patients were varied in relation to comorbidity, Immunization status, and MUAC (Figs. 2, 3, 4 and 5).

### Predictors of time to recovery

Children who had admitted with comorbidity were on average 51% decreased rate of recovery from SCAP as compared with comorbid free children, while holding the other variables in the model constant [AHR = 0.49; 95% CI: 0.32, 0.75)].

Children who reach the hospital after five days of onset of symptoms had a 65% decreased rate of recovery from SCAP as compared with children who reach the hospital within 5 days of onset of symptoms keeping other variables in the model constant [AHR = 0.35; 95% CI: 0.20, 0.60)].

Children having MUAC < = 12.5 cm at admission had a 79% decreased rate of recovery from SCAP as compared with children having MUAC above 12.5 cm at admission while holding other variables in the model constant [AHR = 0.21; 95% CI: 0.12, 0.37)]. Having a smoker who smoke in the house decreased the recovery rate by 79% as compared with children having no smoker in the house while holding other variables in the model constant [AHR = 0.21; 95% CI: 0.10, 0.42)].

Children who were not fully immunized for their age had on average 65% decreased rate of recovery from SCAP as compared with children fully immunized for their age while keeping other variables in the model constant [AHR = 0.35; 95% CI: 0.24, 0.53)] (Table 3).

**Table 3:** predictors of time to recovery from SCAP among 6 to 59 months of age children admitted to south wollo zone public hospitals, North East Ethiopia, 2021. (*n* = 270)

Variable	Categories	Recovered	Not recovered	CHR(95%CI)	AHR(95%CI)
Residence	Urban	121	23	1	1
	Rural	84	42	0.73(0.55,0.96)	1.00(0.65,1.47)
Duration to seek care	<=5days	182	54	1	1
	**> 5days**	**23**	**11**	**0.61(0.39,0.94)**	0.35(0.20,0.60)***
MUAC	**<=12.5 cm**	**19**	**28**	**0.27(0.16,0.43)**	0.21(0.12,0.37)***
	> 12.5 cm	174	32	1	1
Previous history of ALRTI	Yes	61	28	0.67(0.50,0.91)	1.27(0.86,1.87)
	No	144	37	1	1
Comorbidities at admission	**Present**	**72**	**36**	**0.53(0.40,0.71)**	0.49(0.32,0.75)**
	Absent	133	29	1	1
TB	No	95	29	1.25(0.95,1.65)	1.29(0.92,1.82)
	Unknown	110	36	1	1
Anemia	Present	16	12	0.50(0.30,0.83)	1.49(0.79,2.82)
	Absent	189	53	1	1
Convulsion	Present	11	5	0.63(0.34,1.16)	0.50(0.24,1.06)
	Absent	194	60	1	1
Fully immunized for age	Yes	152	36	1	1
	**No**	**53**	**29**	**0.41(0.29,0.56)**	0.35(0.24,0.53)***
Educational status of caregivers	Not able to read and write	63	34	0.72(0.46,0.89)	1.31(0.74,2.32)
	Able to read and write	27	11	0.98(0.57,1.67)	1.31(0.69,2.48)
	Educated (formal education)	88	10	1.22(0.79,1.88)	1.40(0.84,2.33)
	Degree and above	27	10	1	1
Smoker in the house	**Yes**	**11**	**18**	**0.27(0.15,0.50)**	0.21(0.10,0.42)***
	No	194	47	1	1

*Statistically significant at P-value < 0.05, **P-value < 0.01, ***P-value < 0.001

## Discussions

This study tried to assess the time to recovery from severe community-acquired pneumonia and its predictors among 6 to 59 months of age children admitted to south wollo zone public hospitals, in Northeast Ethiopia. The overall recovery rate from SCAP was 20.45 per 100 person-days with a median (IQR) recovery time of 4 (3 to 5) days. The independent predictors like reaching hospitals after 5 days of onset of symptoms, MUAC < = 12.5 cm at admission, having a smoker who smokes in the house, having comorbidities on admission, and being not fully immunized for age were significantly associated with longer periods of recovery time from SCAP.

This study also revealed that the median time to recovery from SCAP was 4 days IQR 3 to 5 days, which is consistent with the findings of the studies conducted at Jimma university specialized hospital [15] And Debre Markos referral hospital [14] which revealed that the estimated median time to recovery from SCAP for all observations was less than 4 days and 4 days respectively. This finding is also almost similar to the study conducted in the rural health center of the Gambia which reported that the meantime of recovery was 4.5 days [18].

The finding of this study is higher than the study done at Vanderbilt (2.3 days) and Nepal (2 days) [19, 20]. This variation might be due to admission criteria, staff and facility setup, and co-morbidity differences [14].

The median time to recovery which is obtained in this study is also much lower than studies conducted in an international population of hospitalized patients with CAP which showed that the time to clinical stability for the majority of patients is 8 days [21], and the study finding in Poland on trends in the hospitalization of children with bacterial pneumonia that reported 8.2 to 10.1 days [22] this difference might be due to case mix and time difference since those studies were conducted before 2015 [14].

Duration prior to seeking care was an independent significant predictor for the recovery time of severs community acquired pneumonia. Children who presented to the hospitals before five days of onset of symptoms recovered earlier than those children presented after 5days. This finding is consistent with a study conducted in Debre Markos referral hospital [14] and prospective study conducted in the Gambia [18]. This might be due to the reason that as children delay to seek care while encountering diseases, the progression of disease increases and making the disease worse and complicated these finally results to delayed time to recovery.

The other important predictor that was significantly associated with recovery time from SCAP was the presence of co-morbidity. Children who were admitted to hospitals with co-morbidity recovered slowly as compared to children who were admitted without co-morbidity. This finding is supported by studies conducted by Jimma [15]and Debre Markos [14]. This might be because encountering many diseases at a time results in impaired immunity in children, which leads to a decreased response to treatment and finally delays the recovery time [11].

Having a smoker in the house is a significant predictor of delayed recovery from SCAP in this study and this finding is consistent with those studies conducted in Jimma [15] and Morocco [16]. This might be due to the reason that smoking including passive smoking is both a cause and an aggravating factor for many respiratory tract diseases which increases Pneumonia severity and thus delayed recovery time [14].

This study also revealed that being not fully immunized for age significantly delayed the recovery time from Severe Community-Acquired Pneumonia. This might be due to the reason that lack of immunization causes increased susceptibility and severity of different infections which delayed recovery time [10].

Having MUAC of less than or equal to 12.5 cm is another significant predictor of delayed recovery in this study. this finding is supported by studies conducted by Debre Markos [14] and Jimma [15]on the association between nutritional status and recovery from severe community-acquired pneumonia both of them showed that there were significant associations between the nutritional status of the child and the status of discharge observed [14, 15]. This might be because of the decreased immunity, increased susceptibility to infection and the occurrence of comorbidities secondary to malnutrition (under nutrition) and their combined effect leads to delayed recovery from illness [15].

## Conclusion and recommendations

Generally, the recovery time of children with SCAP in the study area was within the recommended national standards. Reaching hospitals after 5 days of onset of symptoms, MUAC < = 12.5 cm at admission, having a smoker in the house, presence of comorbidities on admission, and being not fully immunized for age were significantly associated with longer periods of recovery time from SCAP. Measures to shorten recovery time from the disease should be strengthened. Parents or caregivers should take their children to the health facility immediately when they become ill. Health care providers should give due attention to children with the identified predictors while treating them. Further study using a prospective design by including other parental variables that were not included in this study.

## Data Availability

The data used for analysis is fully available in the manuscript file without restriction.

## Abbreviations

AHR: Adjusted Hazard Ratio
AOR: Adjusted Odds Ratio
ARTI: Acute Respiratory Tract Infection
CI: Confidence Interval
DAMA: Discharge against Medical Advice
EBF: Exclusive Breast Feeding
EDHS: Ethiopian Demographic Health survey
FMOH: Federal Ministry of Health
HIV: Human Immuno-deficiency Virus
IMNCI: Integrated Management of Newborn and Childhood Illnesses
NGOs: Non-governmental Organization
PCV: Pneumococcal Conjugate Vaccine
PEM: Protein Energy Malnutrition
SCAP: Severe Community-Acquired Pneumonia
WHO: World Health Organization

## References

[1] Porth C, Gaspard KJ. Essentials of pathophysiology: concepts of altered health states. Wolters Kluwer Philedelphia; 2015.

[2] Higgins-Steele A, Yousufi K, Sultana S, Ali AS, Varkey S. Ending preventable child deaths from pneumonia and diarrhea in Afghanistan: an analysis of intervention coverage scenarios using the lives saved tool. Journal of Tropical Medicine. 2017;2017. 10.1155/2017/3120854 PMC5337376 28298932

[3] Behrman RE, Kliegman RM, Jenson HB. Nelson textbook of pediatrics. Saunders Philadelphia; 2004.

[4] Mackenzie G. The definition and classification of pneumonia. Pneumonia. 2016;8(1):1–5. 28702293 10.1186/s41479-016-0012-z PMC5471962

[5] Geleta D, Tessema F, Ewnetu H. Determinants of community-acquired pneumonia among children in Kersa District, Southwest Ethiopia: facility-based case-control study. J Pediatr Neonatal Care. 2016;5(2):00179.

[6] Kalil AC, Metersky ML, Klompas M, Muscedere J, Sweeney DA, Palmer LB, et al. Management of adults with hospital-acquired and ventilator-associated pneumonia: 2016 clinical practice guidelines by the Infectious Diseases Society of America and the American Thoracic Society. Clin Infect Dis. 2016;63(5):e61–111. 27418577 10.1093/cid/ciw353 PMC4981759

[7] Onyedum CC, Chukwuka J. Admission profile and management of community-acquired pneumonia in Nigeria-5 year experience in a tertiary hospital. Respir Med. 2011;105(2):298–302. 21112756 10.1016/j.rmed.2010.11.003

[8] Selection WECot M, Organization UE. WH. The selection and use of essential Medicines: report of the WHO Expert Committee, 2013 (including the 18th WHO Model List of essential Medicines and the 4th WHO Model list of essential. Medicines for Children): World Health Organization; 2014.

[9] Morris SS, Black RE, Tomaskovic L. Predicting the distribution of under-five deaths by cause in countries without adequate vital registration systems. Int J Epidemiol. 2003;32(6):1041–51. 14681271 10.1093/ije/dyg241

[10] Rudan I, Boschi-Pinto C, Biloglav Z, Mulholland K, Campbell H. Epidemiology and etiology of childhood pneumonia. Bull World Health Organ. 2008;86:408–B16. 18545744 10.2471/BLT.07.048769 PMC2647437

[11] Pan Y, Guan H, Zhou S, Wang Y, Li Q, Zhu T, et al. Initial CT findings and temporal changes in patients with the novel coronavirus pneumonia (2019-nCoV): a study of 63 patients in Wuhan, China. Eur Radiol. 2020;30(6):3306–9. 32055945 10.1007/s00330-020-06731-x PMC7087663

[12] Oestergaard MZ, Inoue M, Yoshida S, Mahanani WR, Gore F, Cousens S, United Nations Inter-Agency Group for Child Mortality Estimation and the Child Health Epidemiology Reference Group, et al. Neonatal mortality levels for 193 countries in 2009 with trends since 1990: a systematic analysis of progress, projections, and priorities. PLoS Med. 2011;8(8):e1001080. 21918640 10.1371/journal.pmed.1001080 PMC3168874

[13] McAllister DA, Liu L, Shi T, Chu Y, Reed C, Burrows J, et al. Global, regional, and national estimates of pneumonia morbidity and mortality in children younger than 5 years between 2000 and 2015: a systematic analysis. Lancet Global Health. 2019;7(1):e47–57. 30497986 10.1016/S2214-109X(18)30408-X PMC6293057

[14] Mengist B, Tesfa M, Kassie B. Time to recovery and predictors of severe community-acquired pneumonia among pediatric patients in Debre Markos referral hospital, North West Ethiopia: a retrospective follow-up study. PLoS ONE. 2020;15(9):e0239655. 32976491 10.1371/journal.pone.0239655 PMC7518609

[15] Bekele F, Sinaga M, Quadri JA, Kumar A, Shariff A, Malik T. Factors associated with outcomes of severe pneumonia in children aged 2 months to 59 months at jimma university specialized hospital, southwest Ethiopia. Curr Pediatr Res. 2017.

[16] Jroundi I, Mahraoui C, Benmessaoud R, Moraleda C, Tligui H, Seffar M, et al. Risk factors for a poor outcome among children admitted with clinically severe pneumonia to a university hospital in Rabat, Morocco. Int J Infect Dis. 2014;28:164–70. 25305555 10.1016/j.ijid.2014.07.027 PMC7129557

[17] Tiewsoh K, Lodha R, Pandey RM, Broor S, Kalaivani M, Kabra SK. Factors determining the outcome of children hospitalized with severe pneumonia. BMC Pediatr. 2009;9(1):1–8. 19236689 10.1186/1471-2431-9-15 PMC2651138

[18] Puchalski Ritchie L, Howie S, Arenovich T, Cheung Y, Weber M, Moore S, et al. Long-term morbidity from severe pneumonia in early childhood in the Gambia, West Africa: a follow-up study. Int J Tuberc Lung Dis. 2009;13(4):527–32. 19335961

[19] Wolf RB, Edwards K, Grijalva CG, Self WH, Zhu Y, Chappell J, et al. Time to clinical stability among children hospitalized with pneumonia. J Hosp Med. 2015;10(6):380–3. 25919391 10.1002/jhm.2370 PMC4456292

[20] Basnet S, Sharma A, Mathisen M, Shrestha PS, Ghimire RK, Shrestha DM, et al. Predictors of duration and treatment failure of severe pneumonia in hospitalized young Nepalese children. PLoS ONE. 2015;10(3):e0122052. 25798907 10.1371/journal.pone.0122052 PMC4370861

[21] Halm EA, Fine MJ, Marrie TJ, Coley CM, Kapoor WN, Obrosky DS, et al. Time to clinical stability in patients hospitalized with community-acquired pneumonia: implications for practice guidelines. JAMA. 1998;279(18):1452–7. 9600479 10.1001/jama.279.18.1452

[22] Gajewska M, Lewtak K, Scheres J, Albrecht P, Gorynski P. Trends in the hospitalization of children with bacterial pneumonia in Poland. Cent Eur J Public Health. 2016;24(3):188. 27755860 10.21101/cejph.a4164

